# A Rare Case of Adenosquamous Carcinoma Arising from Intraductal Papillary Neoplasm of the Bile Duct

**DOI:** 10.70352/scrj.cr.25-0819

**Published:** 2026-06-03

**Authors:** Akifumi Kawahito, Yasuhiro Kihara, Masao Uemura, Junya Toyoda, Hiroshi Yokomizo

**Affiliations:** Department of General Surgery, Japanese Red Cross Kumamoto Hospital, Kumamoto, Kumamoto, Japan

**Keywords:** intraductal papillary neoplasm of the bile duct, adenosquamous carcinoma, cholangiocarcinoma

## Abstract

**INTRODUCTION:**

Intraductal papillary neoplasm of the bile duct (IPNB) is an uncommon biliary tumor that often exhibits invasive growth but generally shows a more favorable prognosis than conventional cholangiocarcinoma. In contrast, adenosquamous carcinoma (ASC) of the bile duct is rare and associated with markedly poor outcomes. We report an extremely rare case of ASC arising from IPNB in the intrapancreatic bile duct.

**CASE PRESENTATION:**

A 78-year-old woman was referred to our hospital for evaluation of jaundice. Contrast-enhanced CT revealed a low-density mass in the pancreatic head, with upstream common bile duct dilatation. Endoscopic retrograde cholangiopancreatography and endoscopic US demonstrated distal bile duct stenosis and a 25-mm hypoechoic mass in the pancreatic head. Biopsy indicated adenocarcinoma, and the lesion was preoperatively diagnosed as pancreatic head cancer involving the distal bile duct. Owing to renal dysfunction, preoperative chemotherapy was not administered, and subtotal stomach-preserving pancreaticoduodenectomy was performed. Histopathological examination revealed high-grade IPNB with invasion into the pancreatic parenchyma. The invasive component demonstrated dual differentiation into adenocarcinoma and squamous cell carcinoma, establishing a final diagnosis of ASC arising from IPNB. Despite curative resection, local recurrence and liver metastases developed 7 months postoperatively, and the patient died 15 months after surgery.

**CONCLUSIONS:**

This is an extremely rare case of ASC presumed to have arisen from IPNB in the intrapancreatic bile duct. Awareness of this pathological progression is essential for accurate diagnosis and prognostic assessment, underscoring the need for meticulous histopathological evaluation.

## Abbreviations

ASC
: adenosquamous carcinoma
IPMN
: intraductal papillary mucinous neoplasm
IPNB
: intraductal papillary neoplasm of the bile duct
SCC
: squamous cell carcinoma
WHO
: World Health Organization

## INTRODUCTION

IPNB is a papillary epithelial tumor arising in the bile ducts and is considered the biliary counterpart of IPMN of the pancreas, as originally described by Chen et al.^1)^ Since its inclusion in the WHO classification more than a decade ago, extensive collaborative studies from East Asia have contributed to a deeper understanding of its clinicopathological characteristics. The incidence of biliary tract tumors in Asia is estimated to be 10%–35%, considerably higher than that in Western countries.^2)^ Surgical resection remains the primary treatment modality, and postoperative outcomes are generally more favorable than those of conventional cholangiocarcinoma; the reported 1-, 3-, 5-, and 10-year overall survival rates of IPNB are 86.9%, 65.8%, 49.8%, and 32.0%, respectively.^3)^

ASC of the bile duct is an uncommon malignancy, accounting for approximately 2% of all cholangiocarcinomas.^4)^ Previous studies have demonstrated that ASC carries a significantly worse prognosis than conventional cholangiocarcinoma.^5,6)^ Histologically, ASC is composed of both glandular and squamous components, although the mechanisms underlying squamous differentiation remain unclear. In the biliary tract, ASC has been proposed to arise *de novo* or through squamous metaplasia within an existing adenocarcinoma; however, its development in association with IPNB has not been documented to date.

Here, we report an extremely rare case of ASC arising from IPNB in the intrapancreatic bile duct. To the best of our knowledge, this is the first case to be reported. We additionally review the relevant literature and discuss potential mechanisms contributing to its histogenesis and unexpectedly aggressive clinical behavior.

## CASE PRESENTATION

A 78-year-old woman was referred to our hospital for further evaluation of jaundice. Physical examination revealed no remarkable findings except for icterus. Laboratory tests showed elevated total bilirubin (10.7 mg/dL) and creatinine (2.06 mg/dL). Serum carcinoembryonic antigen levels were within the normal range (2.0 ng/mL), whereas carbohydrate antigen 19-9 was markedly elevated at 626 U/mL.

Contrast-enhanced CT revealed a low-density mass in the pancreatic head, with upstream common bile duct dilatation on the hepatic side. There was no evidence of distant metastases (**Fig. 1A** and **1B**). Endoscopic retrograde cholangiopancreatography demonstrated a 20-mm stenosis in the distal bile duct and dilatation of the intrahepatic bile ducts, with intraluminal papillary proliferation resembling a tumor embolus. Endoscopic US revealed a 25-mm hypoechoic mass in the pancreatic head with slightly indistinct margins, irregular contours, and internal heterogeneity. Biopsy showed adenocarcinoma (**Fig. 2**). Based on these findings, the lesion was preoperatively diagnosed as pancreatic head cancer with distal bile duct invasion.

**Fig. 1:**
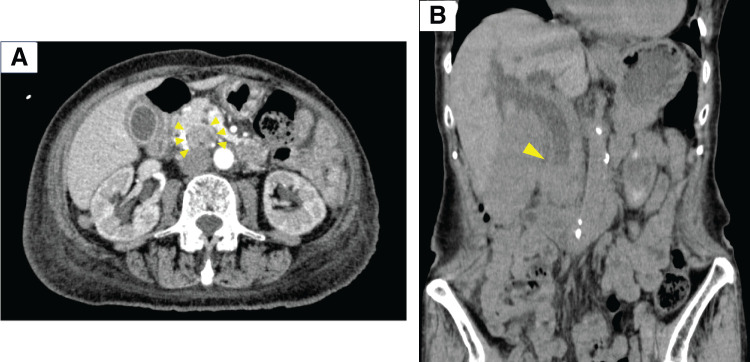
CT findings. (**A**) Contrast-enhanced CT revealed a low-density mass in the pancreatic head (arrowheads, tumor). (**B**) CT revealed upstream common bile duct dilatation on the hepatic side (arrowhead, tumor).

**Fig. 2:**
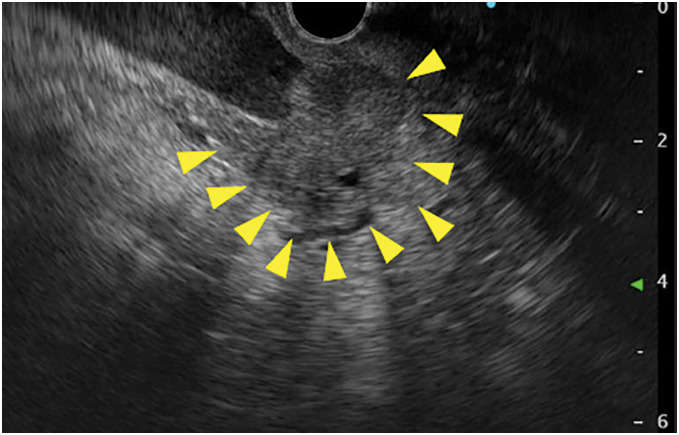
Endoscopic US findings. Endoscopic US demonstrated a 25-mm hypoechoic mass in the pancreatic head with slightly indistinct margins, irregular contours, and internal heterogeneity (arrowheads, tumor).

Because of renal dysfunction, no preoperative chemotherapy was administered. The patient underwent subtotal stomach-preserving pancreaticoduodenectomy with reconstruction using the modified Child method. The operative time was 406 min, and intraoperative blood loss was 100 mL.

Gross examination revealed a 27 × 25-mm papillary tumor located within the intrapancreatic bile duct, continuously infiltrating the pancreas parenchyma. Histopathological evaluation demonstrated a gastric-type high-grade IPNB continuous with the invasive component. The invasive carcinoma exhibited dual differentiation, consisting of approximately 60% adenocarcinoma and 40% SCC. Using immunohistochemistry, we confirmed that the intraluminal IPNB had a direct histological continuity with adenocarcinoma and SCC. Furthermore, SCC was confined to the infiltrative area and did not exist in the intraluminal IPNB lesion. The final diagnosis was ASC arising from IPNB. The tumor invaded the pancreas and duodenum and demonstrated scattered lymphatic invasion, moderate venous invasion, and perineural invasion (**Fig. 3**).

**Fig. 3:**
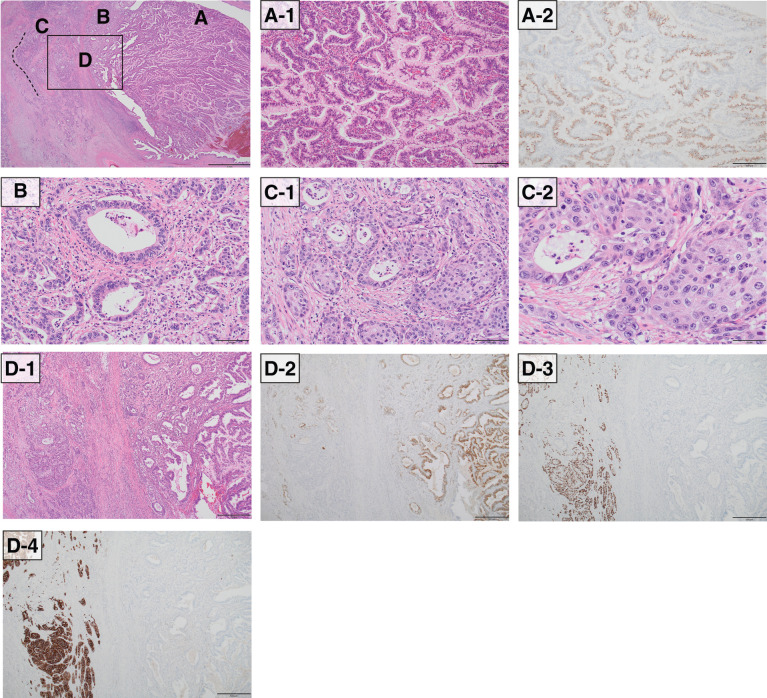
Histopathological findings of the resected specimen. H&E-stained sections of the resected intrapancreatic bile duct. Low-power view (×20): High-grade IPNB infiltrating the intrapancreatic bile duct wall (**A**). Adenocarcinoma was present within the bile duct wall (**B**). The deepest part of the tumor (dashed line) showed ASC invading the pancreatic parenchyma (**C**). IPNB has a direct histological continuity with adenocarcinoma and SCC (**D**). (**A**) High-grade IPNB (**A-1**: H&E, ×100; **A-2**: immunohistochemistry, ×100): IPNB exhibited a highly atypical gastric-type glandular histology. Tumor cells were diffusely positive for MUC5AC. (**B**) Adenocarcinoma in the bile duct wall (H&E, ×200). (**C**) Adenocarcinoma and ASC outside the bile duct (**C-1**: H&E, ×200; **C-2**: H&E, ×400): A gradual transition between glandular and squamous components was observed, including intercellular bridges and sheet-like proliferation, consistent with transformation from adenocarcinoma to SCC. (**D**) A direct histological continuity among IPNB, adenocarcinoma, and SCC (**D-1**: H&E, ×40; **D-2**–**D-4**: immunohistochemistry, ×40): (**D-2**) MUC5AC was positive in the IPNB and showed partial positivity in the adenocarcinoma component in the pancreatic parenchyma; notably, these areas were surrounded by tumor cells showing positivity for (**D-3**) p40 and (**D-4**) CK5/6. Histological continuity is confirmed among IPNB, adenocarcinoma, and SCC, which are arranged sequentially from the bile duct toward the pancreatic parenchyma. ASC, adenosquamous carcinoma; ck, cytokeratin; H&E, hematoxylin–eosin; IPNB, intraductal papillary neoplasm of the bile duct; MUC5AC, mucin 5AC; SCC, squamous cell carcinoma

The postoperative course was complicated by a pancreatic fistula that required drainage for more than 3 weeks, classified as Grade B according to the 2016 definition by the International Study Group of Pancreatic Surgery.^7)^ Seven months after surgery, abdominal CT revealed local recurrence and liver metastases. The patient declined chemotherapy and died 15 months after surgery.

## DISCUSSION

IPNB is defined as an intraductal papillary–villous lesion that is classified into low-grade, high-grade, and associated invasive carcinoma based on the degree of cellular atypia and invasiveness. Histologically, IPNB typically presents as a papillary epithelial tumor with a thin fibrovascular core proliferating within the bile ducts and is categorized into 4 subtypes—pancreatobiliary, intestinal, gastric, and oncocytic—according to morphology and cytological atypia.^2)^ Although IPNB has been regarded as the biliary counterpart of IPMN of the pancreas, important differences exist, including a lower frequency of mucin hypersecretion, broader distribution throughout both intrahepatic and extrahepatic ducts, and distinct epidemiological features.

A new subclassification proposed through joint studies between Japan and Korea and adopted in the fifth edition of the WHO classification divides IPNB into Type I and Type II.^8–10)^ Type I typically arises in the intrahepatic bile ducts, frequently demonstrates excessive mucin production, and commonly belongs to the gastric or oncocytic subtype; approximately 50% are associated with invasive carcinoma. In contrast, Type II lesions occur predominantly in the extrahepatic bile ducts, rarely produce mucin, often exhibit complex histological architecture including solid tubular components, and are associated with invasive carcinoma in more than 90% of cases. Although Type II IPNB has a poorer prognosis than Type I, survival outcomes remain better than those of conventional cholangiocarcinoma. Since the histological subtypes and Type I and Type II classifications do not always correspond, in this case—although the histological subtype was gastric—the lesion exhibited characteristics consistent with Type II IPNB: developing in the intrapancreatic bile duct, exhibiting high-grade dysplasia with invasive carcinoma, and showing minimal mucin production.

ASC of the bile duct is a rare histological subtype characterized by dual differentiation into adenocarcinoma and SCC. According to the WHO classification, ASC of the ampulla of Vater is defined as a tumor containing ≥25% SCC, and its prognosis may be more favorable than ASC arising elsewhere; however, ASC of the bile duct remains poorly understood because of its rarity.^2,4)^ In our case, the invasive component consisted of 60% adenocarcinoma and 40% SCC, with histologic evidence of transition at the invasive front, supporting the hypothesis of progression from adenocarcinoma to SCC.

Several mechanisms have been proposed for ASC histogenesis, including (i) malignant transformation of multipotent epithelial stem cells, (ii) squamous metaplasia of biliary epithelium, (iii) transformation of adenocarcinoma into SCC, and (iv) collision of 2 independent tumors.^11,12)^ Currently, the most widely accepted hypothesis is sequential transformation of adenocarcinoma into SCC, supported by pathological continuity between components.^13)^ Our histopathological findings were consistent with this hypothesis and support a sequential progression from IPNB to adenocarcinoma and ultimately to ASC. Furthermore, SCC was confined to the infiltrative area and did not exist in the intraluminal IPNB lesion, this also suggests that it was not *de novo* SCC.

The prognosis of ASC is generally poor. The reported median survival time is approximately 11 months compared with 32 months for conventional cholangiocarcinoma.^6)^ The aggressive clinical course observed in this patient, characterized by early recurrence despite curative resection, was likely influenced by multiple factors. First, a population-based analysis has shown that biliary ASC tends to present at a more advanced stage and with more frequent regional or distant metastasis than conventional adenocarcinoma.^4)^ Therefore, the presence of a SCC component may have contributed to the unfavorable outcome in this case. Second, the background lesion was classified as Type II IPNB, a subtype that is generally associated with more complex histological features and a higher frequency of invasive carcinoma than Type I IPNB. This classification may also be relevant to the aggressive behavior observed. In addition, established adverse pathological features, including pancreatic parenchymal invasion, lymphatic invasion, and perineural invasion, likely contributed to the poor prognosis.

Taken together, these findings suggest that ASC arising from IPNB may represent a subgroup with unfavorable biological behavior. However, given the rarity of this entity, further accumulation of similar cases will be necessary to clarify whether its prognosis is distinct from that of conventional IPNB-associated invasive adenocarcinoma.

Owing to the rarity of ASC, optimal treatment strategies remain undefined. Surgical resection offers the most favorable prognosis when feasible^4)^; however, postoperative recurrence is common and effective chemotherapy has not been established.^14)^ Some reports have described potential benefit in selected molecular profiles—for example, HER2-positive ASC treated with trastuzumab-based regimens—suggesting opportunities for individualized therapy.^15)^

A PubMed search covering 1980–2024 using the keywords “intraductal papillary neoplasm of the bile duct” and “adenosquamous carcinoma” revealed no previous reports of ASC arising from IPNB, indicating that the present case represents the first documented instance.

## CONCLUSIONS

In summary, we describe an extremely rare case of ASC arising from IPNB. Because the biological behavior, clinicopathological characteristics, and optimal management of ASC associated with IPNB remain poorly understood, further accumulation of cases and comprehensive clinicopathological studies are essential to elucidate its pathogenesis and establish effective treatment strategies.
